# Tetraspanins CD81 and CD82 Facilitate α4β1-Mediated Adhesion of Human Erythroblasts to Vascular Cell Adhesion Molecule-1

**DOI:** 10.1371/journal.pone.0062654

**Published:** 2013-05-21

**Authors:** Frances A. Spring, Rebecca E. Griffiths, Tosti J. Mankelow, Christopher Agnew, Stephen F. Parsons, Joel A. Chasis, David J. Anstee

**Affiliations:** 1 Bristol Institute for Transfusion Sciences, Bristol, United Kingdom; 2 Department of Biochemistry, University of Bristol, Bristol, United Kingdom; 3 Lawrence Berkeley National Laboratory, University of California, Berkeley, California, United States of America; University of Washington, United States of America

## Abstract

The proliferation and terminal differentiation of erythroid progenitors occurs in human bone marrow within erythroblastic islands, specialised structures consisting of a central macrophage surrounded by developing erythroid cells. Many cell-cell and cell-matrix adhesive interactions maintain and regulate the co-ordinated daily production of reticulocytes. Erythroid cells express only one integrin, α4β1, throughout differentiation, and its interactions with both macrophage Vascular Cell Adhesion Molecule-1 and with extracellular matrix fibronectin are critical for erythropoiesis. We observed that proerythroblasts expressed a broad tetraspanin phenotype, and investigated whether any tetraspanin could modulate integrin function. A specific association between α4β1 and CD81, CD82 and CD151 was demonstrated by confocal microscopy and co-immune precipitation. We observed that antibodies to CD81 and CD82 augmented adhesion of proerythroblasts to Vascular Cell Adhesion Molecule-1 but not to the fibronectin spliceoforms FnIII_12-IIICS-15_ and FnIII_12–15_. In contrast, different anti-CD151 antibodies augmented or inhibited adhesion of proerythroblasts to Vascular Cell Adhesion Molecule-1 and the fibronectin spliceoform FnIII_12-IIICS-15_ but not to FnIII_12–15_. These results strongly suggest that tetraspanins have a functional role in terminal erythropoiesis by modulating interactions of erythroblast α4β1 with both macrophages and extracellular matrix.

## Introduction

In normal human bone marrow, terminal erythroid differentiation occurs within erythroblastic islands [1]. This specialised erythropoietic niche, first described by Bessis [2], comprises a central macrophage surrounded by adherent developing erythroblasts. Within islands, extensive cell-cell interactions occur not only between adjacent erythroblasts, but also between erythroblasts and macrophages, such that each erythroblast is in direct contact with macrophage cellular processes [3]. Some of the molecules involved in these intercellular interactions have been identified (reviewed in [1]). These include: i) macrophage sialoadhesin (CD169, Siglec-1) binding to sialylated erythroblast glycoproteins [4], ii) homophilic binding of Erythroblast-Macrophage Protein on both macrophages and erythroblasts [5], iii) macrophage Vascular Cell Adhesion Molecule-1 (VCAM-1) binding to erythroblast α4β1 [6], iv) macrophage αV integrin binding to erythroblast Intercellular Adhesion Molecule-4 [7], and v) macrophage CD163 (receptor for haemoglobin-haptoglobin complexes) binding to an unidentified erythroblast receptor [8].

The importance of α4β1 during erythropoiesis, and of erythroblast α4β1 interactions with macrophage VCAM-1 has been extensively studied. In vivo administration of anti-α4 antibody rendered mice anaemic [9], while in vitro addition of antibodies reactive with anti-α4 or anti-VCAM-1 antibodies reduced stromal cell-dependent erythropoiesis [10] and disrupted erythroblastic island integrity [6]. Additionally, a requirement for appropriately activated α4β1 for the in vitro reformation of erythroblastic islands has also recently been demonstrated in SWAP-70-deficient mice [11]. SWAP-70, a protein involved in integrin regulation and cytoskeletal F-actin rearrangement, affects development of erythroid progenitors in bone marrow and spleen by negative regulation of α4β1 [11]. In normal human bone marrow, α4β1 is clustered at contact sites between macrophages and erythroblasts [12], and this heterophilic cell contact enhances proliferation [5], [13], [14]. A role for α4β1 in the optimal expansion and differentiation of erythroid cells in bone marrow, rather than an absolute requirement of α4β1 in erythropoiesis was also evident in α4-null chimeric mice [15]. Studies of the effects on erythropoiesis of α4, β1 or VCAM-1 deficiencies in different mouse models have yielded conflicting results, and demonstrated different effects in bone marrow and splenic erythropoiesis [15]–[20]. However while conditional knockout mice were not anaemic, a role for α4 and β1 but not for VCAM-1 has been demonstrated in stress erythropoiesis with defects in erythroid progenitor expansion in bone marrow and/or spleen, and in cell maturation [11], [18]–[20].

The continued expression of α4β1, the only integrin expressed throughout terminal erythroid maturation [21], [22], suggests that interactions within erythroblastic islands between erythroblast α4β1 and its ligands, macrophage VCAM-1 and fibronectin [23], are both important for effective erythropoiesis. The early erythroid progenitors, BFU-E and CFU-E, and preproerythroblasts, adhere to fibronectin via both integrins α4β1 and α5β1 [21], [24], [25]. Whereas α5β1 expression is lost on basophilic erythroblasts, the continued expression but progressive down-regulation of α4β1 during terminal maturation is accompanied by a progressive decrease in attachment to fibronectin until the reticulocyte stage, where these cells are non-adherent [25]. While fibronectin has only one binding site for α5β1, there are five sites for α4β1, three in alternatively spliced regions [26]. The temporal expression of α4β1 and α5β1 during differentiation and the complex expression of fibronectin spliceoforms in adult bone marrow [27] hint at distinct and stage-specific functions for integrin/fibronectin interactions during erythroid proliferation and differentiation. Indeed, fetal liver erythroblast α4β1 interaction with fibronectin is essential for maximal erythroid expansion [28]. The appropriate activation state of α4β1 is also important for α4β1-fibronectin interactions since SWAP-70-deficient CFU-E hyper-adhere to fibronectin in vitro [11].

Many membrane proteins, including integrins, are components of multi-molecular complexes that together regulate their interactions and functions [29]–[32]. It has recently been suggested that erythroblast membrane proteins may also associate in complexes [14] since antibodies to any one protein disrupts macrophage-erythroblast interactions and island integrity [4], [6]–[8], [33]. Integrins, including α4β1, are found in complexes with tetraspanins in various cell types [29], [34]. The tetraspanins are a large family of small, widely expressed cell surface proteins that interact with a broad range of proteins such as other tetraspanins, integrins, Immunoglobulin Superfamily proteins and other adhesion molecules, ectoenzymes and intracellular signalling molecules [35], [36]. Tetraspanins associate through lateral interactions with other tetraspanins and membrane proteins to form tetraspanin-enriched microdomains [36]. By organising multimolecular membrane complexes they regulate many cellular processes including modulating ligand binding, adhesion strengthening, cell migration, proliferation, cell fusion and signalling events [35]–[38]. Tetraspanins also localize to intracellular vesicles, suggesting a role in protein trafficking [29]. The observations that tetraspanins CD81 (Target of Anti-Proliferative Activity-1) and CD151 (Platelet-Endothelial Tetra-span Antigen-3) are associated with α4β1 and can augment cell adhesion to fibronectin in erythroleukemic cells [39]–[41] led us to investigate the hypothesis that tetraspanins were also associated with α4β1 in primary erythroblasts and could play a role in erythropoiesis by regulating erythroblast α4β1 interactions with macrophage VCAM-1 and/or fibronectin.

This report describes the first complete tetraspanin profile of human erythroblasts derived in vitro from peripheral blood CD34+ progenitors in suspension culture. We show that erythroblasts express several tetraspanins found on other haematopoietic cells and continue to express CD81, CD82 and CD151 on terminally differentiating cells. Focusing on three stages of late maturation, the proerythroblast, basophilic erythroblast and polychromatic erythroblast, we demonstrate a physical and functional association between CD81, CD82 and CD151 with α4β1 by confocal microscopy, co-immune precipitation and cell attachment assays to α4β1 ligands, VCAM-1 and fibronectin. We show that from the proerythroblast through to later stages of erythroid maturation a proportion of plasma membrane α4β1 molecules are associated with both CD81- and CD82-enriched microdomains, and that CD81 and CD82 associate with each other. Furthermore, antibodies to both CD81 and CD82 augment proerythroblast attachment to VCAM-1, but have little effect on more mature cells, or on attachment to fibronectin spliceoforms, FnIII_12-IIICS-15_ (H/120) and FnIII_12–15_ (H/0). CD151 also associates with α4β1 but anti-CD151 could either augment or inhibit proerythroblast adhesion to both VCAM-1 and fibronectin spliceoform H/120, but not to H/0. These data strongly suggest that CD81, CD82 and CD151 play an important role during erythropoiesis by modulating the adhesive properties of α4β1.

## Materials and Methods

### Ethics Statement

Buffy coats, a waste fraction from anonymous donations of platelets by apheresis, were provided with written informed consent for research use in accordance with both the Declaration of Helsinki and with the policy of the National Health Service Blood and Transplant. The research into the mechanisms of in vitro erythropoiesis was reviewed and approved by the Southmead Local Research Ethics Committee 08/05/2008 REC number 08/H0102/26.

All reagents, tissue culture media, growth factors, cytokines and certain antibodies were purchased from Sigma (Poole, Dorset, UK) unless stated otherwise.

### Erythroid Cultures

Human CD34+ haematopoietic progenitor cells were isolated from human blood donor mononuclear cells (waste buffy coats, see Ethics Statement) by positive selection using the MiniMACS magnetic bead system (Miltenyl Biotech, Bisley, UK) as described by the manufacturer’s protocol. The isolated CD34+ cells were pooled and cultured at 37°C in a humidified atmosphere of 5% CO_2_ in air in a 2-stage protocol to induce differentiation along the erythroid lineage following a modified method of Griffiths et al. [42]. CD34+ cells were seeded at 1.5×10^5^/ml in primary medium and maintained at 2×10^5^/ml after day 3. When the first glycophorin A (GPA)-positive cells appeared (days 5–6) erythroblasts were harvested, washed thrice in Hanks Balanced Salt solution and seeded into secondary culture medium and maintained at 3–8×10^5^/ml. Protocol A used Iscove’s Modified Dulbecco Medium (IMDM) containing 1% (w/v) bovine serum albumin (BSA), 10 µg/ml recombinant human insulin and 200 µg/ml iron saturated human transferrin (“Stemspan SFEM”, Stemcell Technologies SARL). In the first stage of culture (day 0–5 or 6) the IMDM was supplemented with 10 ng/ml recombinant human (rH) Stem Cell Factor (SCF, R & D Systems Europe, Abingdon, Oxfordshire, UK), 3 U/ml Erythropoietin (Epo, Roche Products, Welwyn Garden City, UK), 1 ng/ml rH Interleukin-3 (IL-3, R & D Systems Europe), 1 µl/ml cholesterol-rich lipid mix, 0.1 ng/ml Prograf (Fuisawa, Killorglin, Ireland) and 1 U/ml penicillin and streptomycin. In the second stage (days 5–6 onwards) the primary medium was replaced with Stemspan SFEM supplemented with additional 800 µg/ml iron saturated human transferrin, 3% v/v heat inactivated human male group AB serum, 10 U/ml Epo, 10 ng/ml insulin, 1 mM tri-iodothyroxine and 1 U/ml penicillin and streptomycin. Protocol B primary culture medium (days 0–5) comprised IMDM (Source BioScience) containing 3% male AB serum, 2% foetal bovine serum (Hyclone, Fisher Scientific UK Ltd), 200 mg/ml iron-saturated transferrin, 10 ng/ml SCF, 3 U/ml Epo, 1 ng/ml IL-3, 0.1 ng/ml Prograf and 1 U/ml penicillin and streptomycin. Secondary stage medium (day 5 onwards) comprised IMDM, 3% male AB serum, 2% foetal bovine serum, 300 mg/ml iron-saturated transferrin, 3 U/ml Epo, 10 ng/ml insulin, 0.1 ng/ml Prograf and 1 U/ml penicillin and streptomycin. Cultures grown in Protocol B media showed increased proliferation over those grown in Protocol A media.

### HEL Cell Line

The human erythroleukemia line, HEL, (ECACC, Salisbury, Wiltshire, UK) was maintained at 3×10^5^/ml in Iscove’s Modified Dulbecco’s Medium (IMDM, PAA, Laboratories GmbH, Pasching, Austria) containing 10% foetal bovine serum (Fetal Clone 1, HyClone, Logan, Utah) at 37°C in a 5% CO_2_ atmosphere.

### Flow Cytometry

Antigen expression was analyzed by flow cytometry as described [43]; antibodies used are listed in Table 1. RPE-conjugated isotype negative control antibodies were from eBioscience Ltd (Hatfield, Hertfordshire, UK) and BioLegend UK Ltd (Cambridge, Cambridgeshire, UK). Secondary antibody RPE F(ab’)_2_ goat anti-mouse IgG Fc-specifc was from Dako Cytomation (Glossop, UK). After labelling the cells were fixed in phosphate buffered saline (PBS) containing 1% (v/v) BSA (Lorne Laboratories, Reading, Berkshire, UK) and 1% w/v paraformaldehyde then analysed on a FC500 flow cytometer using Kaluza software (Beckman Coulter (UK) Ltd, High Wycombe, Buckinghamshire, UK).

**Table 1:** Antibodies used for flow cytometry and confocal microscopy.

Specificity	RPE-labelled clones	Source	Unlabelled clones	Source
CD9	MM2/57	AbD Serotec^1^	MM2/57, Gi15	AbD Serotec^1^ Alexis Corporation^2^
CD37	424925	R&D Systems^3^	M-B371, NMN-46	BD Biosciences^4^ Invitrogen^5^
CD53	HI29 425514	BioLegend^6^ R&D Systems^3^	MEM-53, 65-5A3	AbD Serotec^1^ Alexis Corporation^2^
CD63	MEM-259	AbD Serotec^1^	MEM-259, H5C6	AbD Serotec^1^ BD Biosciences^4^
CD81	5A6, 454720	BioLegend^6^ R&D Systems^3^	JS81, 1D6	BD Biosciences^4^ AbD Serotec^1^
CD82	ASL-24, B-L2	BioLegend^6^ AbCam^7^	TS82, B-L2	AbCam^7^ AbD Serotec^1^
CD151	IIG5a, 210127	AbD Serotec^1^ R&D Systems^3^	IIG5a, 210127	AbD Serotec^1^ R&D Systems^3^
α4, CD49d	44H6, 9F10	AbD Serotec^1^ eBioscience^8^	HP2/1, Max68P	AbD Serotec^1^ Dr T Shock^9^
α5, CD49e	238307, P1D6	R&D Systems^3^ eBioscience^8^	IIA1, JSB5	BD Biosciences^4^ AbD Serotec^1^
αL, CD11a	mab38, TS2/4	AbD Serotec^1^ BioLegend^6^	TS1/22, mab38	ATCC^10^ AbD Serotec^1^
αIIb, CD41	PM6/248, PAB-1	AbD Serotec^1^ IBGRL^11^	PAB-1, 5B12	IBGRL^11^
GPA	BRIC256	IBGRL^11^	BRIC256	IBGRL^11^
Kell	BRIC68	IBGRL^11^	BRIC18, BRIC68	IBGRL^11^IBGRL^11^
AE-1			BRIC6, BRIC200	IBGRL^11^
Control	mG1	eBioscience^8^	mG1	Sigma^12^
Control	mG2a	BD Biosciences^4^	mG2a	Sigma^12^
Control	mG2b	AbD Serotec^1^	mG2b	Sigma^12^
Control	mG3	eBioscience^8^	mG3	Sigma^12^

1 AbD Serotec, Oxford, Oxfordshire, UK.

2 Alexis Corporation, Enzo Life Sciences (UK) Ltd, Exeter, Devon, UK.

3 R&D Systems, Abingdon, Oxfordshire, UK.

4 BD Biosciences, Oxford, Oxfordshire, UK.

5 Invitrogen Ltd, Paisley, UK.

6 BioLegend UK Ltd, Cambridge, Cambridgeshire, UK.

7 AbCam, Cambridge, Cambridgeshire, UK.

8 eBioscience Ltd, Hatfield, Hertfordshire, UK.

9 A gift from Dr T Shock, CellTech Chiroscience, Slough, UK.

10 American Type Culture Collection USA.

11 IBGRL Reseach Products, Bristol, UK.

12 Sigma, Poole, Dorset, UK.

### Confocal Microscopy

Cells (3×10^5^ cells per coverslip) were seeded on 0.01% (w/v) poly-L-lysine coated coverslips and incubated for 30 minutes at 37°C in 5% CO_2_. Cells harvested from culture on days 5 to 10 were fixed with 3% paraformaldehyde (TAAB, Aldermaston, UK) for 20 minutes and permeabilised with 0.05% (w/v) digitonin (Merck Millipore, Beeston, Nottinghamshire, UK) for 5 minutes, then incubated for 15 minutes in 4% BSA (Park Scientific, Northampton, Northamptonshire, UK). Subsequent washes were carried out in PBS. Cells harvested from culture from day 11 onwards were fixed in 1% paraformaldehyde and permeabilised in 0.05% saponin (Merck Millipore). Subsequent washes and antibody dilutions were carried out in PBS containing 0.005% saponin, 5 mg/ml BSA and 1 mg/ml glucose. When dual labelling with two monoclonal primary antibodies the first antibody was subjected to an extra conversion step by the addition of AffiniPure Fab fragment rabbit anti-mouse IgG (Jackson ImmunoResearch, Stratech Scientific Ltd, Newmarket, Suffolk, UK) at a concentration of 1/20 in 4% BSA. After this conversion step the second monoclonal antibody was added. Primary antibodies used are listed in Table 1. Goat anti-mouse Alexa fluor® 488 or goat anti-rabbit Alexa fluor® 546 (Invitrogen, Carlsbad, California, USA) conjugated secondary antibodies were diluted in 4% normal goat serum and incubated with the cells for 30 minutes at room temperature in the dark. Coverslips were mounted on Vectashield® Mounting Medium (Vector Laboratories, Burlingame, California, USA) on microscope slides and sealed with nail varnish. Samples were imaged at 22°C using 40× oil immersion lenses (magnification = 101.97 µm at zoom 3.8, numerical aperture 1.25) on a Leica SP5 confocal imaging system. Images were obtained using Leica software and subsequently processed using Adobe Photoshop.

### Immune Precipitation, SDS-PAGE and Immunoblotting

Cells were washed thrice in 10 mM Tris, 150 mM NaCl, pH 7.4 (TBS), containing either 2 mM ethylenediaminetetraacetic acid (EDTA), 1 mM CaCl_2_ plus 1 mM MgCl_2_ or 1 mM MnCl_2_ (TBS +/− cations/EDTA) by centrifugation at 400 g for 5 mins at 4°C. The cell pellet was lysed for 25 mins on ice in lysis buffer (TBS +/− cations or 2 mM EDTA, 1% (w/v) polyoxyethylene (10) oleyl ether (Brij-97), 2 mM phenyl methyl sulphonyl fluoride (PMSF) and EDTA-free protease inhibitor cocktail (Complete™, Roche Diagnostic GmbH, Mannheim, Germany). In some experiments polyoxyethylene (20) oleyl ether (Brij-99), Triton X-100 (TX-100), Nonidet P40 (NP40) or 3-[(3-cholamidopropyl)dimethylammonio]-1-propane sulphonate hydrate (CHAPS) were also used at 1% w/v in place of Brij-97. Cells were solubilised at 10^7^/ml for HEL cells and proerythroblasts (days 5–6 of culture), 1.5×10^7^/ml for basophilic erythroblasts (days 7–8 of culture) and 3×10^7^/ml for polychromatic erythroblasts (days 11–12 of culture), centrifuged at 208,000 g for 30 mins at 4°C and the supernatants were stored at −80°C. Immune precipitates were isolated from the cell lysates with antibodies (see Table 2) pre-coupled to Protein G Sepharose 4 Fast Flow (PGS, GE Healthcare Life Sciences, Little Chalfont, Buckinghamshire, UK) after an initial pre-clearing step with Protein G-coupled isotype control antibodies [40]. Immune complexes were eluted into non-reduced sample buffer [44] containing 2 mM PMSF and EDTA-free protease inhibitor cocktail. Samples were run on 7.5% or 12% (w/v) non-reduced polyacrylamide gels. Immunoblotting under semi-dry transfer conditions was as described [7] except that PVDF membranes (Millipore UK Ltd, Watford, Hertfordshire, UK) were blocked with PBS containing 0.8% v/v Tween-20 and 5% w/v γ-globulin-free BSA fraction V. Biotinylated antibodies to CD81 (clone 1.3.3.2, Alexis Corporation, Enzo Life Sciences (UK) Ltd, Exeter, Devon, UK) and rabbit anti-sera to α5, β1, β2 and β3 (R&D Systems, Abingdon, Oxfordshire, UK) were used. Rabbit monoclonal anti-α4 antibody was from Cambridge Bioscience (Cambridge, Cambridgeshire, UK), polyclonal anti-CD82 antibody was from AbCam (Cambridge, Cambridgeshire, UK) and anti-CD53 (MEM-53), anti-CD63 (MEM-259) and anti-CD151 (IIG5a) antibodies were from AbD Serotec. Secondary reagents were streptavidin-alkaline phosphatase conjugate (Perkin Elmer, Cambridge, Cambridgeshire, UK) and alkaline phosphatase-conjugated F(ab’)_2_ fragments of goat anti-sera to rabbit and mouse IgG (Jackson ImmunoResearch). Membranes were developed with Western Lightning CDP Star Chemiluminescent Reagent (Perkin Elmer) and images were recorded on a Kodak imager using Kodak imaging software.

**Table 2:** Source, epitope location and functional properties of antibodies used for immune precipitation and adhesion assays.

Specificity	Clone	Source	Epitope	Functional effects
CD37	NMN46	Invitrogen^1^		Blocking
CD53	MEM-53	AbD Serotec^2^	1	Increases intracellular Ca^2+^
CD53	63-5A3	Alexis Corp^3^	1	
CD53	HI29	BD Bioscience^4^	Carbohydrate	Yes
CD63	MEM-259	AbD Serotec^2^		
CD63	H5C6	BD Bioscience^4^	1	
CD63	TEA3/18	Abnova^5^	1	Yes
CD81	1D6	AbD Serotec^2^	A	Aggregating
CD81	JS81	BD Bioscience^4^	C	Non-functional
CD81	454720	R&D Systems^6^		
CD81	3.3.1.2	Alexis Corp^3^		
CD82	TS82b	AbCam^7^		
CD82	53H5	eBioscience^8^		T cell activation
CD82	423524	R&D Systems^6^		
CD82	ASL-24	BioLegend^9^		
CD82	B-L2	AbD Serotec^2^		
CD151	IIG5a	AbD Serotec^2^		
CD151	50-6	eBioscience^8^		Blocks in vivo metastasis
CD151	210127	R&D Systems^6^		
β1, CD29	TS2/16	ATCC^10^	βA domain, αα 207-218	Activating
β1, CD29	mab13	BD Bioscience^4^	βA domain, αα 207-218	Blocking
Α4, CD49d	HP2/1	AbD Serotec^2^	β propeller, 3^rd^ repeat, αα 195-268	Blocking
Α5, CD49e	IIA1	BD Bioscicence^4^		Blocking
αL, CD11a	TS1/22	ATCC^10^	αA domain	Blocking
αIIb, CD41	PAB-1	IBGRL^11^		
Controls	Murine	Sigma^12^		
Controls	Rat	eBioscience^8^		

1 Invitrogen, Paisley, UK.

2 AbD Serotec, Oxford, Oxfordshire, UK.

3 Alexis Corporation, Enzo Life Sciences (UK) Ltd, Exeter, Devon, UK.

4 BD Biosciences, Oxford, Oxfordshire, UK.

5 Abnova, Novus Europe Ltd, Cambridge, Cambridgeshire, UK.

6 R&D Systems, Abingdon, Oxfordshire, UK.

7 AbCam, Cambridge, Cambridgeshire, UK.

8 eBioscience Ltd, Hatfield, Hertfordshire, UK.

9 BioLegend UK Ltd, Cambridge, Cambridgeshire, UK.

10 American Type Culture Collection USA.

11 IBGRL Reseach Products, Bristol, UK.

12 Sigma, Poole, Dorset, UK.

### Fusion Proteins

7-domain Vascular Cell Adhesion Molecule-1-Fc fusion protein (VCAM-Fc) was purchased from R&D Systems. Human fibronectin constructs FnIII_12–15_ (H/0) and FnIII_12-IIICS-15_ (H/120), in the pGEX expression vector, were a kind gift from Prof. M. Humphries (University of Manchester) [45]. The constructs were transformed into Rosetta 2(DE3) pLysS E. coli cells (Novagen, Merck Millipore) for expression as previously described [46]. In brief, 10 ml Luria-Bertani (LB) broth cultures with 50 µg/ml carbenicillin and 35 µg/ml chloramphenicol, were inoculated from a glycerol stock and grown overnight at 37°C. 1 L of LB media was inoculated and grown to OD600 = 0.6. Protein expression was induced by addition of 1 mM isopropyl β-D-1-thiogalactopyranoside at 20°C for 16 h. Cells were harvested by centrifugation, the cell pellet was resuspended in buffer A (50 mM tris pH 8, 150 mM NaCl) supplemented with a protease inhibitor tablet (Roche Diagnostics) and DNAseI. The protein was released by sonication and the insoluble fraction pelleted by centrifugation. The supernatant was loaded onto a 5 mL GSTrap FF column (GE Healthcare, Little Chalfont, Buckinghamshire, UK) pre-equilibrated with buffer A. Recombinant protein was eluted by an increasing gradient of buffer B (50 mM tris pH 8, 150 mM NaCl and 10 mM reduced glutathione). The protein was further purified by gel filtration on a HiLoad 16/60 superdex 200 (GE Healthcare) equilibrated in buffer A. Pooled recombinant protein fractions were analysed by SDS-PAGE for >95% purity and confirmed by N-terminal sequencing and mass-spectrometry. Liberation of recombinant human fibronectin H/0 from the glutathione synthase (GST)-tag was accomplished by incubation at 4°C for 20 h with 25 U thrombin. The sample was applied to a GSTrap FF column and benzamidine column (GE Healthcare) in tandem to remove cleaved GST-tag, uncleaved protein and thrombin. Cleaved recombinant H/0 was further purified by gel filtration as above.

### Cell Adhesion Assays

Attachment assays were performed essentially as described [47] with modifications. Immulon-4 HBX 96 well plates (Dynex Technologies, Billingshurst, UK) were coated with either 1 µg/ml goat anti-human Fc or anti-GST antibody (AbCam) in bicarbonate buffer overnight at 4°C then incubated with either Fc- or GST-fusion proteins as appropriate overnight at 4°C. Fibronectin H/0 fragment without the GST tag was coated directly onto the plates in PBS. Plates were blocked with PBS containing 4% fraction V BSA. Media for fluorescently labelling cells were either RPMI1640 containing 0.1% BSA (RPMI LB) or IMDM containing 0.1% BSA and 2 mM ethylene glycol tetracetic acid (IMDM LB). Activation of cells with 80 µM phorbol myristate acetate (PMA) was performed at the same time as fluorescence labelling in IMDM LB. Cells were washed after labelling in either RPMI LB or IMDM LB supplemented with additional CaCl_2_ plus MgCl_2_ to 1 mM of each, or with 1 mM MnCl_2_ or 10 mM MgCl_2_ (assay buffers, AB). Fluorescently labelled cells in AB were added at 2×10^5^/well for proerythroblasts (day 5 of culture), 2.5×10^5^/well for basophilic erythroblasts (days 7/8 of culture) and 3×10^5^/well for polychromatic erythroblasts (day 11–12 of culture). Titration assays had 4 replicates per dilution while 6 replicates were used in antibody activation/inhibition assays. For the latter assays, cells were incubated for 15 mins at room temperature in AB containing 10 µg/ml antibodies before addition to the plates. The plates were coated with proteins at a concentration where just less than maximal cell attachment was obtained. The statistical software package, SigmaPlot12, was used for the one way analysis of variation of results of adhesion assays performed in the presence of added antibodies.

## Results

### Tetraspanin and Integrin Expression during Erythropoiesis

**Figure 1:**
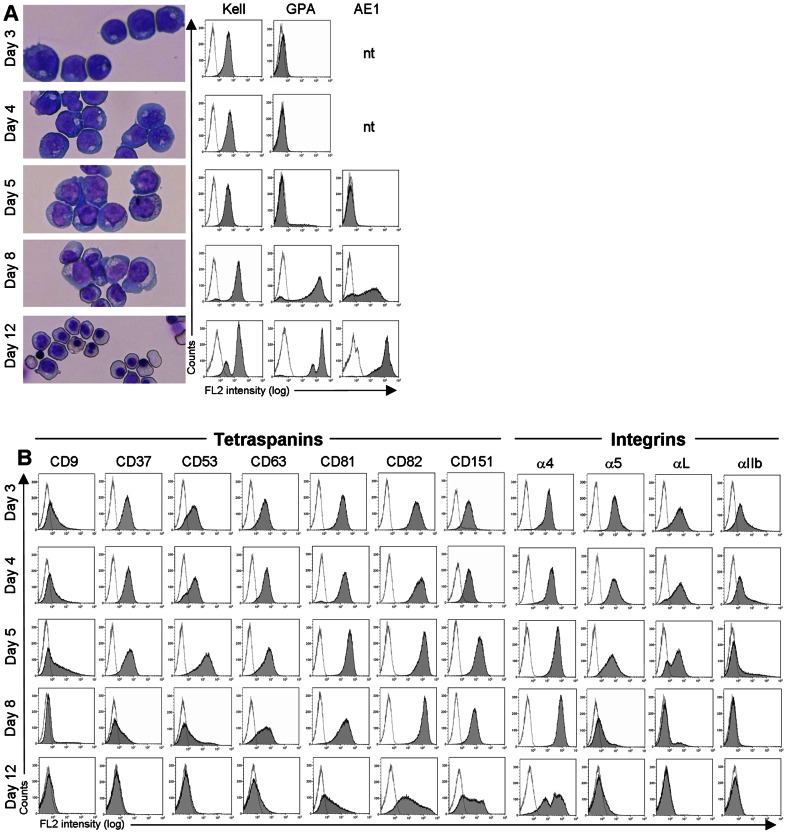
Erythroid culture characterisation and expression of tetraspanins and integrins during terminal maturation. A. Temporal expression of erythroid-specific markers, Kell, GPA and AE1 and morphology of the culture at the same time points. AE1 was tested from day 5 onwards. B. Tetraspanin and integrin profile of the same cultures as shown in A. Results are depicted from one culture where directly conjugated antibodies were used (days 3 and 4) and a second culture with indirectly labelled antibodies (day 5 onwards). The y-axis scale is linear to 350 counts; the x-axis is logarithmic to 10^4^. Images were captured on a Leica DM750 microscope, x20 magnification, using Image-Pro Express 6.0 software.

In our 2-stage cultures 100% of cells expressed the Kell glycoprotein (Kell) by day 3, glycophorin A (GPA) first appeared about day 5 while the erythroid anion exchanger-1 (AE1) was expressed after day 7 (Figure 1A), consistent with the temporal expression of these proteins [48]. The cultures were synchronous at day 5 comprising 90–95% proerythroblasts, predominantly (80%) basophilic erythroblasts on day 8, and and a mixed population of polychromatic and orthochromatic erythroblasts, with a minority of reticulocytes and free nuclei by day 12 (Figure 1A). We looked at the expression of 7 haematopoietic tetraspanins in these cultures and observed that from day 3, when all cells expressed Kell, until the first appearance of GPA (around day 5), approximately 45% of cells expressed CD9 whereas all cells were positive for CD37, CD53, CD63, CD81, CD82 and CD151. This tetraspanin profile is similar to that reported for CD34+ cells and the leukaemic proerythroblast cell line, HEL [49], [50]. At later stages of maturation, CD9 was down-regulated first (almost negative by day 8, basophilic erythroblasts), followed by CD37 and CD53 (negative by day 12, polychromatic erythroblasts), and lastly, CD63 and CD81 (weaker expression by day 12). CD82 and CD151 were still expressed on day 12, consistent with their continued expression at low levels on mature erythrocytes [51], [52]. Throughout erythroid maturation CD82, and to a lesser extent CD81, consistently showed the highest levels of expression of all tetraspanins.

Erythroblasts also showed developmentally regulated expression of 4 integrins (Figure 1B), consistent with previous reports [21], [22], [53]. High levels of α4 were found throughout the culture period (days 3–12), with lower levels of α5, αL and αIIb at the early stages of culture (days 3–5). There was no or very weak expression of αV or other β1 family integrins after day 5 (data not shown). It is interesting to note that early GPA-negative erythroid cells (pre-proerythroblasts and proerythroblasts) express four integrins (α4, α5, αL and αIIb) and their full complement of 7 tetraspanins (CD9, CD37, CD53, CD63, CD81, CD82 and CD151) while more mature GPA+ erythroblasts express CD81, CD82, CD151 and α4β1 and down-regulate expression of other tetraspanins and integrins.

### CD81 and CD82 Colocalise with α4β1 throughout Erythroid Maturation

**Figure 2:**
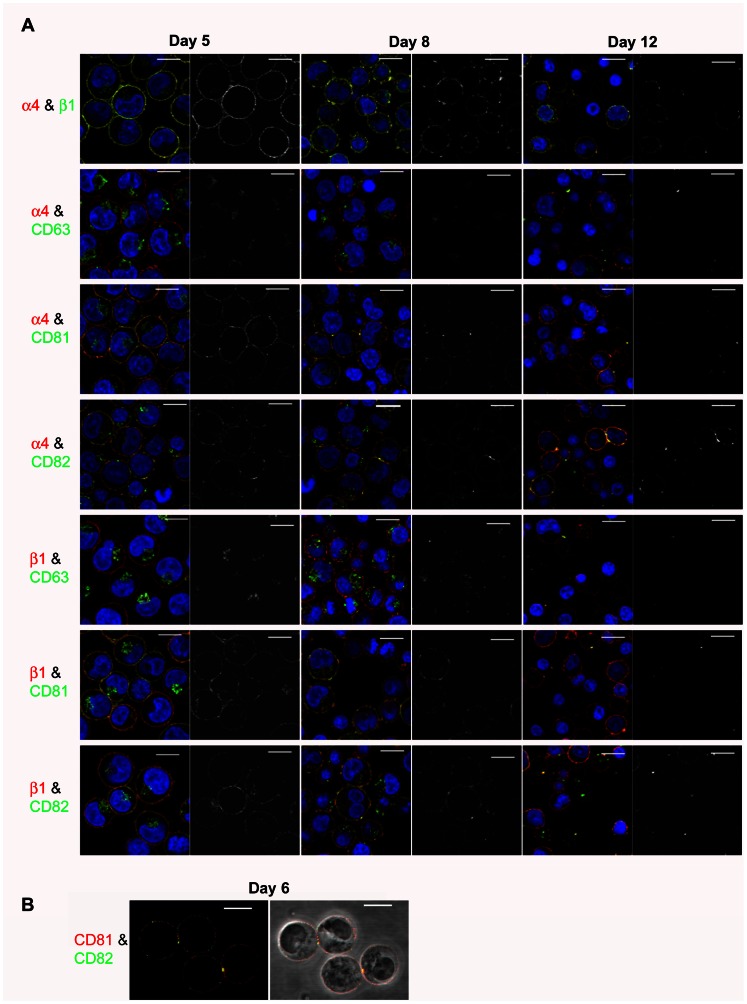
Confocal imaging of erythroblasts. A. (A) Dual staining of α4 (red) and β1 (green) (top panel) and of tetraspanins CD63, CD81 and CD82 (all green) with α4 and β1 integrins (both red) on days 5, 8 and 12 of culture. Colocalisation is seen in yellow on the coloured images and the adjacent grayscale images highlight yellow areas of colocalisation only (scale bars = 10 µm). (B) Cell surface staining of CD81 (red) and CD82 (green) on day 6 erythroblasts shown in fluorescence and phase contrast. Colocalisation is seen in yellow on the both the coloured and phase images (scale bars = 10 µm).

**Figure 3:**
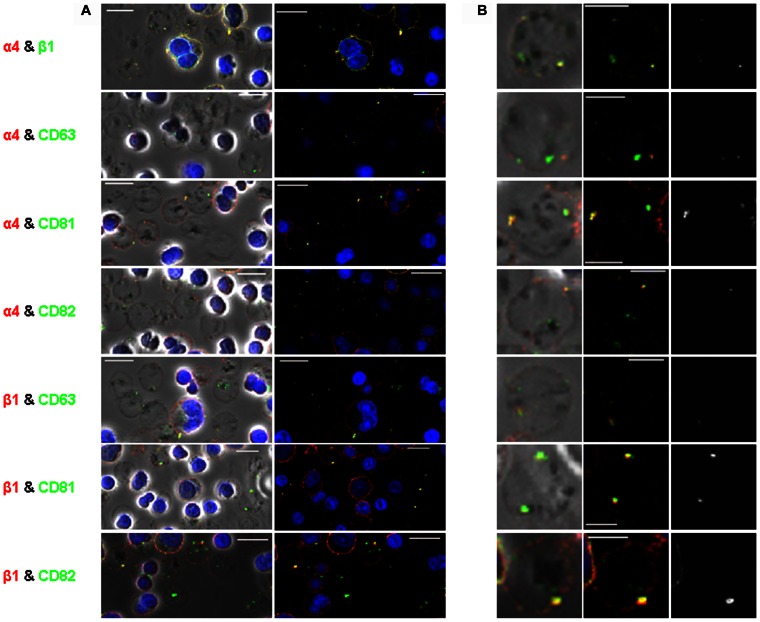
Confocal imaging of reticulocytes. A. (A) Dual staining of tetraspanins CD63, CD81 and CD82 (all green) with α4 and β1 integrins (both red) on day 12 of culture shown in phase contrast and fluorescence (scale bars = 10 µm). Colocalisation is seen in yellow. (B) Single examples of reticulocytes zoomed in from images in (A) shown in phase contrast, fluorescence and greyscale images. Yellow on phase contrast and fluorescence denotes colocalisation. The righthand grayscale images highlight yellow areas of colocalisation only (scale bars = 5 µm).

To explore whether CD81 and/or CD82 associate with α4β1 during erythropoiesis, we performed dual immunofluorescence staining of α4 and β1 subunits with these tetraspanins at 3 time points during terminal differentiation (days 5, 8 and 12, proerythroblasts to reticulocytes). We also examined colocalisation with CD63, which is not reported to be associated with α4β1 but is found released in exosomes with α4β1, and with CD151 which is expressed on red cells [51]. CD151 was not pursued as the antibody clone was poor by immunofluorescence and only showed internal staining on day 6 (Figure S1) and became progressively weaker as the cells matured (data not shown). α4 colocalised with β1 at the cell surface at all 3 time points, although the distribution of colocalisation changed as the cells matured (Figure 2A). On day 5 (proerythroblasts), α4 and β1 were present together in small discrete microdomains; by day 8 (basophilic erythroblasts) these areas of colocalisation appeared to coalesce into larger aggregates less evenly spread over the cell surface; by day 12 (polychromatic and orthochromatic erythroblasts) these large aggregates were still evident with weaker staining on reticulocytes (Figure 2A). Tetraspanins CD81 and CD82 followed this pattern of cell surface colocalisation with both α4 and β1, with fewer but larger microdomains apparent as the cells matured (Figure 2A). By day 12, there was a heterogenous population of enucleating erythroblasts and reticulocytes present (Figure 3). CD81 and CD82 continued to colocalise with both α4 and β1 in late nucleated erythroblasts in a few large vesicles (Figure 3A). The coalescence of the integrin- and tetraspanin-containing vesicles was even more apparent after enucleation, with staining present in a few large vesicles in reticulocytes, with CD82 staining more abundant than CD81 (Figure 3B). Only at the reticulocyte stage was CD63 also found colocalised at the cell surface with both α4 and β1 on day 12, although usually in one large vesicle (Figure 3B). We hypothesise that these α4β1-positive vesicles coated with CD63, CD81 and CD82 are about to be released from early reticulocytes as exosomes [42], resulting in the loss of the majority of CD63, CD81 and α4β1 and a proportion of CD82. This is consistent with the removal of murine β1 by the exosome pathway [54] and findings that low levels of CD82 but not α4β1, CD63 or CD81 are present on mature erythrocytes [52].

We also observed some colocalisation of CD81 and CD82 in small discrete and unevenly distributed cell surface microdomains in day 6 proerythroblasts (Figure 2B). This pattern of colocalisation differed from the more even distribution of the α4β1-CD81 and α4β1-CD82 complexes described above. Interestingly, colocalisation of CD81 with CD82 was particularly evident at areas of cell contact, suggesting the involvement of tetraspanins in intererythroblast interactions within erythroblastic islands.

### CD81, CD82 and CD151 Coprecipitate α4β1 and αIIbβ from Erythroblasts

**Figure 4:**
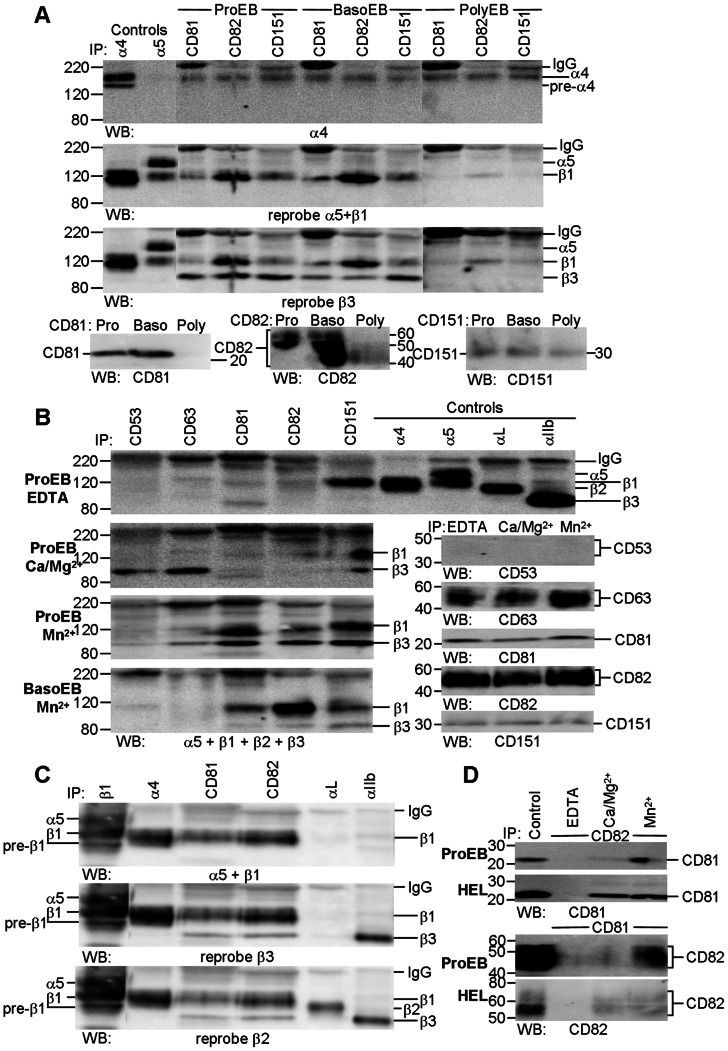
Tetraspanins CD81, CD82 and CD151 are associated with α4β1 throughout erythroid maturation and with β3 in proerythroblasts and basophilic erythroblasts. A. CD81, CD82 and CD151 precipitates from Mn^2+^-activated proerythroblasts (ProEB, day 5), basophilic (BasoEB, day 8) and polychromatic (PolyEB, day 12) erythroblasts were successively probed with anti-α4, anti-β1 and anti-β3 antibodies; tetraspanin controls from each time point are also illustrated. All tetraspanins co-precipitated α4 and β1 from erythroblasts B. Tetraspanin precipitates from day 6 proerythroblasts (ProEB) solubilised in the presence of EDTA or different cations, and from Mn^2+^-activated basophilic erythroblasts (BasoEB, day 8) were probed with a mix of antibodies to α5, β1, β2 and β3 integrins while the control samples were probed with the relevant tetraspanin antibodies. For clarity, integrin controls are illustrated for the EDTA blot but were present on all blots. β1 and β3 integrins were precipitated well only in the presence of Mn^2+^. C. CD81 and CD82 precipitates from day 5 proerythroblasts were successively probed with different anti-integrin subunit antibodies and demonstrate co-precipitation of β1 and β3 but not α5 or β2 integrins. D. CD81 (454720) and CD82 (53H5) precipitates from day 6 proerythroblasts (ProEB) and HEL cells (HEL) solubilised in the presence of EDTA, Ca^2+^+Mg^2+^ or Mn^2+^ probed with anti-CD82 and anti-CD81 antibodies. Each tetraspanin co-precipitates the other most strongly in the presence of Mn^2+^ from proerythroblasts while any cation permits co-precipitation in HEL cells. Integrins were analysed on 7.5% gels, tetraspanins on 12% gels; non-reducing conditions. Unless stated, the following clones were used: CD53, MEM-53; CD63, MEM-259; CD81, 454720; CD82, TS82b; CD151, IIG5a; α4, HP2/1; α5, IIA1; αL, TS1/22; αIIb, PAB-1. All day 5 and 6 cultures comprised 90–95% proerythroblasts; day 8 culture comprised 5% proerythroblasts, 81% basophilic erythroblasts and 14% polychromatic erythroblasts; day 12 culture comprised 41% polychromatic erythroblasts, 15% orthochromatic erythroblasts and 41% reticulocytes. In the day 5 and 6 cultures 15–34% of cells were GPA+ and 28–35% of cells were αIIb+. Day 8 and day 12 cultures had 77% and 97% GPA+ cells, respectively, and 9% and 0% αIIb+ cells, respectively.

Preliminary experiments with the leukemic proerythroblast cell line, HEL, showed the optimum conditions for co-precipitation of β1 by anti-CD81, anti-CD82 and anti-CD151 antibodies occurred after solubilisation in Brij-97 with Mn^2+^ (Figure S2) and subsequent experiments with primary cells used this detergent. Antibodies to the three tetraspanins co-precipitated both α4 and β1 from proerythroblasts (ProEB, day 5) and basophilic erythroblasts (BasoEB, day 8), and more weakly from polychromatic erythroblasts (PolyEB, day 12, Figure 4A). Only the mature fully glycosylated integrins were co-precipitated by the anti-tetraspanin antibodies; both immature and mature glycosylated α4 was evident in control samples. β3 integrin was also co-precipitated by the three anti-tetraspanin-specific antibodies from β3-expressing cells (proerythroblasts and basophilic erythroblasts). Since basophilic erythroblasts expressed very little αIIb, most of the integrin may be from an intracellular pool rather than a cell surface expressed pool. All anti-CD81 (1D6, JS81, 1.3.3.2 and 454720), anti-CD82 (TS82b, 53H5, ASL-24, 423524 and B-L2) and anti-CD151 (IIG5a, 50–6 and 210127) co-precipitated α4β1 and β3 from normal and leukemic proerythroblasts (Figure S3 A–C). Good co-precipitation of β1 and β3 integrins by anti-CD81 and anti-CD82 antibodies and of β3 by anti-CD151 antibodies was only observed when cells were activated by Mn^2+^ (Figure 4B, S4). The β3 signal intensity was very strong in HEL, correlating with high expression of αVβ3 and αIIbβ3 in this cell line (Figures S2–S4). In contrast, co-precipitation of β1 by anti-CD151 antibodies was cation independent (Figure 4B, ProEB; Figure S4), suggesting a stronger association of α4β1 with CD151 than with other tetraspanins. The association of α4β1 with tetraspanins CD81, CD82 and CD151 was a specific interaction since anti-CD53 and anti-CD63 antibodies did not co-precipitate β1 after activation with Mn^2+^ and only co-precipitated β3 well in physiological concentrations of Ca^2+^ and Mg^2+^ (Figure 4B). Sequential probing of immunoblots of CD81 and CD82 precipitates from proerythroblasts with antibodies to α5β1, β3 and β2 clearly demonstrated the specific association between tetraspanins CD81 and CD82 and with β1 and β3 integrins and little or no association with α5 or β2 integrins (Figure 4C). In addition to their association with integrins, tetraspanins also associate with each other [37], so we explored the CD81–CD82 association and its cation dependency. We observed a stronger association between CD81 and CD82 in the presence of Mn^2+^ when compared with Ca^2+^ and Mg^2+^ in primary proerythroblasts (Figure 4D, ProEB). HEL cells differed somewhat in that any cation supported reciprocal co-precipitation of CD81 and CD82 (Figure 4D, HEL). These precipitation data reinforce the confocal studies described above, and clearly demonstrate the association of tetraspanins CD81 and CD82 with one another, and with activated α4β1 during terminal erythroid maturation. We also showed a very strong association between CD151 and α4β1 throughout maturation, and a strong association of CD81, CD82 and CD151 with β3 integrins in HEL cells and in primary proerythroblasts and basophilic erythroblasts.

### Vascular Cell Adhesion Molecule-1 and Fibronectin Fragment FnIII_12-CSIII-15_ are High Affinity Ligands for α4β1 while Binding to Low Affinity Fibronectin Fragment FnIII_12–15_ is Optimal in Activated Basophilic Erythroblasts

**Figure 5:**
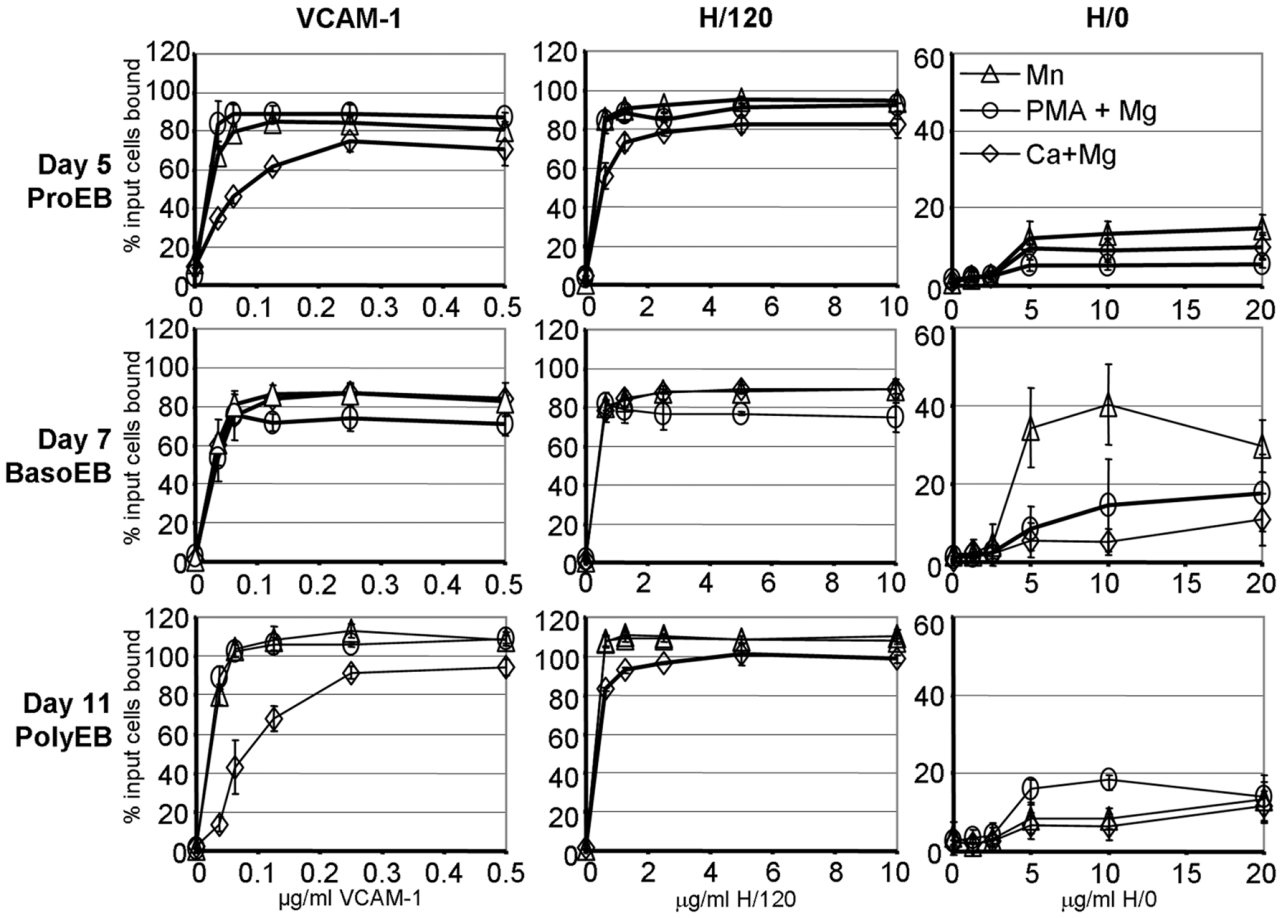
Effect of different activation conditions on erythroblast α4β1 attachment to Vascular Cell Adhesion Molecule-1 and fibronectin fragments, H/120 and H/0. Erythroblasts were allowed to attach to dilutions of VCAM-1, H/120 and H/0 in the presence of different cations to activate α4β1. ◊, 1 mM Ca^2+^ plus 1 mM Mg^2+^; Δ, 1 mM Mn^2+^; ○, 80 µM PMA plus 10 mM Mg^2+^. Day 5 cells (proerythroblasts, ProEB) were from one culture while days 7 (basophilic erythroblasts, BasoEB) and 11 (polychromatic erythrobalsts, PolyEB) cells were from a second culture which was also used for the assays depicted in (Fig. 6) on subsequent days. Each data point is the mean of 4 replicates with the ± standard deviation errors bars shown. Readings in excess of 100% input cells bound were only evident in day 11 cells. High levels of haemoglobin within the cells quenches the fluorescence of the initial 100% input cells bound reading, and is evident with highly activated cells; this artefact does not occur with non-haemoglobinised day 5 cells.

To explore the interaction of α4β1 with VCAM-1 and fibronectin throughout terminal maturation, we performed static adhesion assays using several different integrin activating conditions. The 7-domain VCAM-1 construct contains two α4β1-binding sites, in domains one and four. We used two fibronectin spliceoforms found in human bone marrow [27], FnIII_12–15_ (H/0) and FnIII_12-CSIII-15_ (H/120). H/0 is the lowest affinity ligand for α4β1 [55] and consists of a PRARI motif in the B–C loop of domain 14 [45]. H/120 has three α4β1 attachment sites, the PRARI motif and two sites in the complete alternatively spliced IIICS domain, inserted between domains 14 and 15 [26]. Within this IIICS domain the highest affinity site in the CS1 region comprises the LDV motif, while the REDV motif in the CS5 region has a lower affinity for α4β1. VCAM-1 supported high levels of cell attachment at a 10-fold lower coating concentration than H/120 and H/0 under all activating conditions at the three time points (Figure 5) suggesting that VCAM-1 was the highest affinity ligand for erythroblasts at all stages of maturation. H/120 also supported similarly high maximal levels of cell attachment at all stages of maturation. The lowest levels of adhesion to both VCAM-1 and H/120 were seen with physiological concentrations of Ca^2+^ with Mg^2+^ at the proerythroblast (ProEB, day 5) and polychromatic (PolyEB, day 11) stages. Activation with either Mn^2+^ or with PMA plus Mg^2+^ increased the affinity of attachment to both ligands at both time points. In contrast, adhesion of cells at the basophilic stage (BasoEB, day 7) differed from other time points since there was little difference in the binding affinity of cells to VCAM-1 and H/120 in the presence of Ca^2+^ and Mg^2+^, or with Mn^2+^, while lower affinity binding was evident after activation with PMA plus Mg^2+^. H/0, when captured as a GST-fusion protein, did not support erythroblast adhesion at any time point (data not shown) although low levels of attachment were evident when the protein without the GST tag (pure H/0) was coated directly onto the plate (Figure 5). This suggests that a specific conformation of H/0 is required for cell attachment, which is not maintained in the H/0-GST fragment. While pure H/0 did not support more than 20% of input cell binding under any activating conditions at the proerythroblast and polychromatic stages (days 5 and 11), different results were again obtained with basophilic stage cells (day 7). Although activation by PMA plus Mg^2+^ had a minor effect on cell attachment, Mn^2+^ greatly increased the binding affinity of basophilic cells to pure H/0. The diverse effects of the different integrin activation conditions on basophilic erythroblast attachment to the three α4β1 ligands strongly suggests that the integrin undergoes developmentally regulated changes that alter its ligand binding profile during terminal maturation.

### Anti-CD81 and Anti-CD82 Antibodies have a Pro-adhesive Effect on Proerythroblast Adhesion to Vascular Cell Adhesion Molecule-1 while an Anti-CD151 Antibody Augments Proerythroblast Adhesion to both Vascular Cell Adhesion Molecule-1 and Fibronectin Fragment FnIII12-CSIII-15

**Figure 6:**
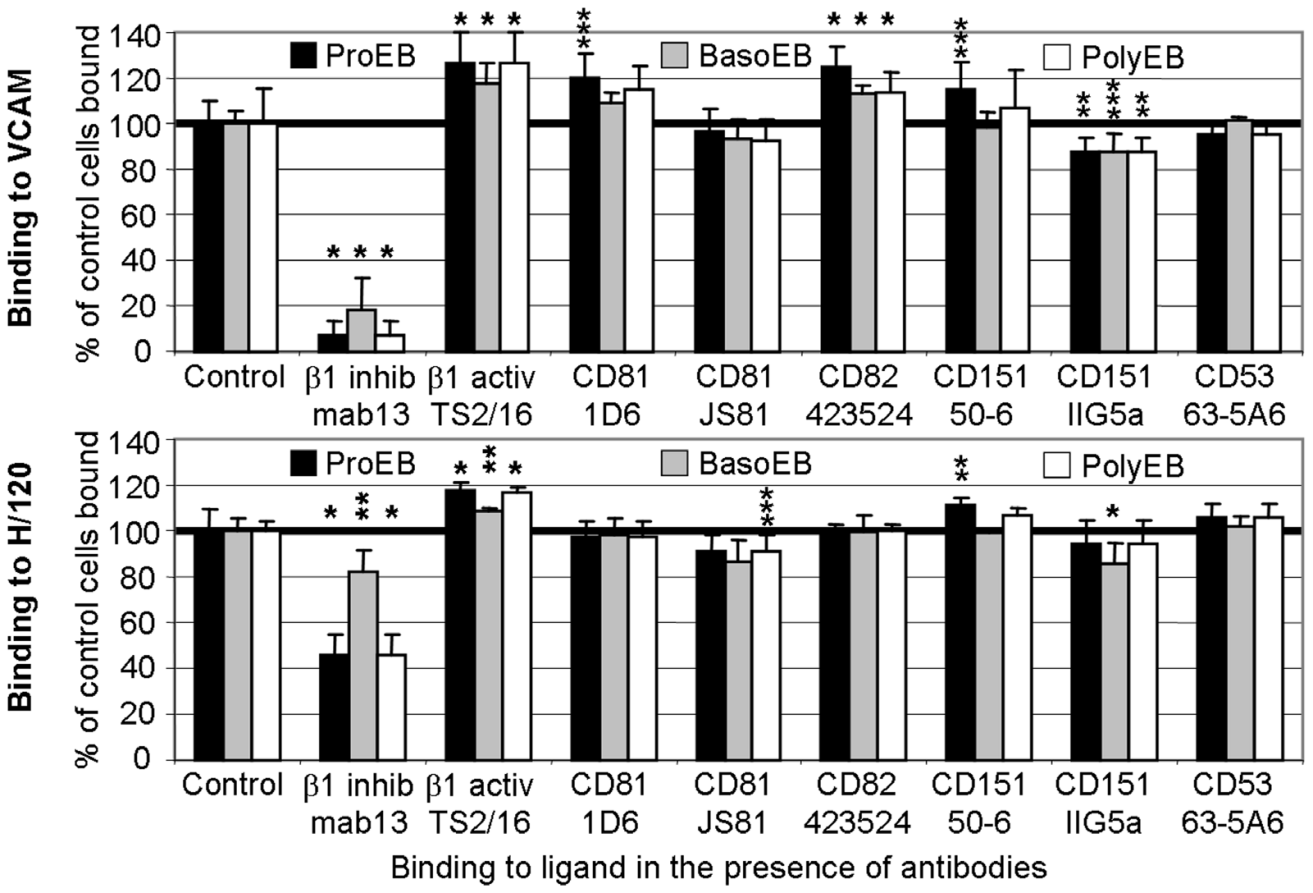
Effect of anti-tetraspanin antibodies on erythroblast attachment to Vascular Cell Adhesion Molecule-1 and fibronectin fragment H/120. Attachment of erythroblasts to ligand in the presence of 1 mM Ca^2+^ plus 1 mM Mg^2+^ and 10 µg/ml of isotype control, inhibitory or activating anti-β1 and anti-tetraspanin antibodies. Each data point is the mean of 6 replicates, each expressed as the percentage of the average of the relevant isotype control cells bound (the normalised values); standard deviations are shown, calculated from the normalised values. The results depicted are from the same culture at 3 time points (ProEB, day 5; BasoEB day 8; PolyEB, day 12) and are representative results of the series of data collected. *, P<0.001; **, P<0.050; ***, P = 0.050–0.055 compared with the relevant isotype control values as determined by one way analysis of variation. Box-Whisker plots of the complete series of experiments performed with CD81, CD82 and CD151 clones with both ligands, with statistically significant results highlighted, are depicted in Figures S5 and S6. Altogether 6 cultures were assayed (days 5 and 8 were performed on 5 occasions, day 12 on 4 occasions). The pre-coating concentrations of VCAM-1 and H/120 allowed slightly less than maximal cell attachment. VCAM-1 was pre-coated at 0.2 µg/ml, 0.125 µg/ml and 0.25 µg/ml while H/120 was pre-coated at 2 µg/ml, 1.25 µg/ml and 1 µg/ml for proerythroblasts (ProEB, black bars, day 5), basophilic erythroblasts (BasoEB, grey bars, day 8) and polychromatic erythroblasts (PolyEB, white bars, day 12) respectively. Readings in excess of 100% input cells bound were sometimes evident in haemoglobinised cells (days 8 and 12). High levels of haemoglobin within the cells quenches the fluorescence of the initial 100% input cells bound reading, and was evident only with day 12 cells in this assay (all the H/120 results and only the VCAM-1 with TS2/16 result); this artefact does not occur with non-haemoglobinised day 5 cells. Day 5 culture comprised 5% pre-proerythroblasts, 91% proerythroblasts and 4% basophilic erythroblasts (28% GPA+); day 8 culture comprised 11% proerythroblasts, 60% basophilic erythroblasts and 28% polychromatic erythroblasts (88% GPA+); day 12 culture comprised 15% basophilic erythroblasts, 48% polychromatic erythroblasts, 15% orthochromatic erythroblasts and 21% reticulocytes (99% GPA+).

Using static adhesion assays we investigated whether adhesion of erythroblasts to VCAM-1, H/120 and H/0 in physiological concentrations of Ca^2+^ with Mg^2+^ was affected by anti-tetraspanin antibodies. Attachment to both VCAM-1 and H/120 was augmented by 108–127% in the presence of the β1-activating mAb, TS2/16, and was lowest on basophilic erythroblasts (118% and 108% respectively, BasoEB, Figure 6). Adhesion to both ligands was inhibited to a certain degree by the anti-β1-inhibitory mab 13 antibody at the 3 time points tested although inhibition was less marked on basophilic erythroblasts, and more effective against VCAM-1 than against H/120 (93%, 82% and 93% inhibition as compared to 54%, 18% and 54% inhibition, respectively, Figures 6, S5, S6). Neither anti-β1 antibody affected the low levels of attachment to H/0 in the presence of Ca^2+^ and Mg^2+^ at any time point (data not shown), despite evidence for increased integrin affinity induced by Mn^2+^ at the basophilic stage (BasoEB, figure 5).

Two anti-CD82 antibodies (423524 and TS82) and the anti-CD81 antibody 1D6 (epitope A), but not other anti-CD81 antibodies (JS81 [epitope B] and 454720) augmented proerythroblast adhesion to VCAM-1 by 115–125%, a similar level to that seen for the activating anti-β1 antibody (Figure 6, ProEB, Figure S5 and data not shown). In contrast, no anti-CD81 or anti-CD82 antibody had any effect on erythroblast adhesion to H/120 or H/0 at any stage of maturation (H/120, Figures 6, S6; H/0 data not shown). We also observed effects with anti-CD151 antibodies on adhesion to VCAM-1 and H/120 but not to H/0. The functional clone, 50-6, augmented proerythroblast but not more mature erythroblast adhesion to both VCAM-1 and H/120, whilst a minor inhibitory effect on VCAM-1 binding was also seen with IIG5a at all stages of erythroid maturation (Figures 6, S5, S6). Other anti-tetraspanin antibodies to CD151 (210127), CD37 (MNM46), CD53 (63-5A3) and CD63 (TEA3/18, MEM-259) had minimal effects on adhesion to VCAM-1, H/120 or H/0 at any time point (Figure 6 and data not shown).

## Discussion

Our report is the first detailed description of the tetraspanin profile of primary human erythroblasts. Proerythroblasts expressed seven tetraspanins concomitantly with four integrins, while more differentiated cells expressed only α4β1 together with tetraspanins CD81, CD82 and CD151, all of which are known to associate with α4β1 in other hemopoietic cells, including CD34+ and HEL cells [39]–[41], [56], [57]. Confocal imaging demonstrated the cell surface colocalisation of discrete pools of α4β1 with both CD81 and CD82, and of CD81 with CD82 throughout differentiation. The distribution of the cell surface α4β1-CD81-CD82 microdomains changed with increasing erythroblast maturation, suggesting a reorganisation of proteins within the plasma membrane. As the cells matured these complexes appeared to amalgamate, becoming fewer but larger in size. Evidence for an α4β1-CD81-CD82 complex throughout late stage maturation was also demonstrated by co-precipitation. Protein association was dependent on the presence of divalent cations, particularly Mn^2+^, an observation not reported for other cells [39], [58], [59]. Our data suggest that more stable α4β1-CD81-CD82 microdomains assemble when erythroblast α4β1 is in a highly activated state.

We also demonstrated that the association of CD81 and CD82 with α4β1 was functionally significant, since antibodies to both tetraspanins augmented proerythroblast adhesion to VCAM-1 in the presence of physiological concentrations of Ca^2+^ with Mg^2+^. We did not use Mn^2+^ in these assays as it has recently been suggested that augmentation effects of tetraspanins on integrin affinity are only evident in systems where conditions are not optimal [38]. There is little evidence for effects of tetraspanin antibodies on the affinity of integrin-mediated adhesion to ligands in static adhesion assays in other cells [29], or on integrin-extracellular matrix protein interactions [37]. Instead, effects are mainly seen in post-ligand binding events, and are evident in integrin-dependent cell spreading, motility and morphology [29], [35], [37]. In this context CD81 enhanced α4β1-mediated adhesion strengthening to stromal cell fibronectin [60] and to VCAM-1 under shear flow [61]. Similarly, CD9 induced pre-B cell adhesion to bone marrow fibroblast-bound fibronectin by up-regulating the avidity of α4β1 and α5β1 [62]. Our data suggest that CD81 and CD82 can increase the affinity of α4β1 for VCAM-1 perhaps by promoting receptor clustering. The functional effects of the anti-tetraspanin antibodies suggest that both tetraspanins modulate proerythroblast-macrophage interactions.

Tetraspanin CD151 was also physically and functionally associated with α4β1, irrespective of activation state, in contrast to CD81 and CD82. Different anti-CD151 antibodies also had consistently different functional effects not only on adhesion to VCAM-1, but also to the fibronectin H/120 fragment. Similar to data obtained for tetraspanins CD81 and CD82, the pro-adhesive effect was again evident at the proerythroblast stage of maturation. While the ability of CD151 to regulate integrin-mediated adhesion strengthening in other cells has been extensively studied [35], this is the first report of an effect on α4β1-mediated adhesion. Our data suggest that tetraspanin CD151 also modulates α4β1-mediated erythroblast binding not only to macrophages, but also to fibronectin, the latter finding also seen in HEL cells [40]. These observations suggest that CD151 could modulate early proerythroblast interactions with several ligands. Indeed, minor effects of CD151 on erythropoiesis have been noted in both CD151-null individuals and one mouse model [51], [63]. CD151 may also be important for megakaryopoiesis since anti-CD151 decreases megakaryocyte progenitor generation in stromal cell cultures [57]. We also observed that tetraspanins CD81, CD82 and CD151 were associated with activated β3 integrins in primary and leukemic erythroblasts, suggesting that these proteins may similarly also modulate early proerythroblast-fibronectin interactions, perhaps affecting erythroid progenitor proliferation and/or differentiation.

We found that the ligand preference for erythroid α4β1 is VCAM-1>H/120>H/0, and this high affinity interaction with VCAM-1 is similar to other hemopoietic cells [64], [65]. The activation states attainable by α4β1 and its ligand profile are cell type-specific and regulated by unknown factors [64]. Moreover, functionally distinct pools of α4β1 exist together in the membrane, the low affinity pool regulating α4β1-mediated adhesion [65]. Several of our observations also suggested a developmentally regulated alteration in ligand binding and in the activation states attainable by α4β1 in erythroid cells (Figure 5). There was a change in adhesion to H/0 and only basophilic erythroblast attachment to H/0 was activated by Mn^2+^, with no effect on proerythroblast or polychromatic stage cells (Figure 5). Similarly the different cations activated basophilic erythroblast attachment to VCAM-1 and H/120 to the same extent, in contrast to the differences seen with these cations on proerythroblast and polychromatic erythroblast attachment. Furthermore, there was a reduced ability of mab 13 to inhibit basophilic erythroblast attachment to H/120 when compared with inhibition of attachment of proerythroblasts and polychromatic erythroblasts (Figure 6, Figure S6). Since mab 13 recognises an epitope that is attenuated by both VCAM-1 and H/120 binding to α4β1 [66], our results suggest that developmentally regulated changes of basophilic erythroblasts α4β1 increase the ability of H/120 to displace mab 13. Our attachment assays suggest that for proerythroblasts and also perhaps for pre-proerythroblasts, the association of α4β1 with CD81, CD82 and CD151 increases the affinity and/or clustering of α4β1 and promotes erythroblast/macrophage interactions, in preference to the erythroblast α4β1-fibronectin interaction. An anti-CD151 antibody also augmented erythroblast-fibronectin interactions, suggesting that the association of CD151 with α4β1 can additionally promote proerythroblast-extracellular matrix interactions. Our results demonstrate that tetraspanins can modulate specific α4β1-ligand interactions, in contrast to the reported overall general negative regulation of all erythroblast α4β1 ligand interactions by SWAP-70 [11]. Newham et al., [66] have suggested that as different ligands induce different conformational changes in α4β1, ligand-specific signals can be transduced into the cell. In the context of erythroid cells, this could result in different down-stream signalling events after either fibronectin or VCAM-1 engagement, and may promote the effective erythroblast proliferation and differentiation programme when cells attach to macrophage VCAM-1 and develop within erythroblastic islands.

## Supporting Information

Figure S1 **CD151 fluorescence in day 6 erythroblasts.** CD151 showed internal fluorescence (green) in day 6 erythroblasts but was negative by fluorescent confocal microscopy as the cells matured (data not shown).
(TIF)

Figure S2 **Several anti-tetraspanin antibodies co-precipitate β1 integrins from HEL cells solubilised in Brij-97.** Precipitates were prepared from HEL cells solubilised in different detergents in the presence of Mn^2+^. CD53, MEM-53; CD63, MEM-259; CD81, 454720; CD82, TS82b; CD151, IIG5a; α4, HP2/1; αL, TS1/22; αIIb, PAB-1. Precipitates were run on 7.5% non-reduced gels.
(TIF)

Figure S3 **All anti-CD81, anti-CD82 and anti-CD151 clones co-precipitate β1 and β3 integrins from normal and leukemic proerythroblasts.** A. CD81 clones. B. CD82 clones. C. CD151 clones. ERB, day 6 proerythroblasts. α4, HP2/1; α5, IIA1; αL, TS1/22; αIIb, PAB-1. Integrins in 7.5% NR gels, tetraspanins in 12% non-reduced gels. More β3 integrins are co-precipitated from HEL cells as they express αIIbβ3 and αVβ3; proerythroblasts express only αIIbβ3.
(TIF)

Figure S4 **Co-precipitation of β1 and β3 integrins by tetraspanins with different cations from HEL cells.** Precipitates were prepared from HEL cells solubilised in Brij-97 in the presence of EDTA or cations. β1 is co-precipitated by CD63, CD81, CD82 and CD151 while β3 is co-precipitated by all tetraspanins in the presence of Mn^2+^. CD151 co-precipitates β1 under all conditions. Integrins were separated on 7.5% gels, tetraspanin controls on 12% gels, both non-reducing conditions. CD53, MEM-53; CD63, MEM-259; CD81, 454720; CD82, TS82b; CD151, IIG5a; α4, HP2/1; αL, TS1/22; αIIb, PAB-1.
(TIF)

Figure S5 **Effect of antibodies to β1 and tetraspanins on the binding of erythroblasts to Vascular Cell Adhesion Molecule-1.** Box-Whisker plots of normalized values (calculated from the average value of isotype control cells bound) of each data point from all experiments performed at three stages of maturation, with the median, 10^th^, 25^th^, 75^th^ and 90^th^ percentiles depicted as vertical boxes with error bars. *, P<0.001, **, P<0.050; ***, P = 0.051, compared with the relevant isotype control values for each clone as determined by one way analysis of variance (which is a subset of all control values depicted at each time point). All antibodies were tested at each time point at least three times except for CD82 423524 (twice on days 5 and 8, once on day 12) and CD81 1D6 (once on day 12). Coating concentrations of VCAM-1Fc were 0.2 µg/ml, 0.125 µg/ml and 0.25 µg/ml for ProEB, BasoEB and PolyEB, respectively.
(TIF)

Figure S6 **Effect of antibodies to β1 and tetraspanins on the binding of erythroblasts to fibronectin fragment FnIII_12-IIICS-15_.** Box-Whisker plots of normalized values (calculated from the average value of isotype control cells bound) of each data point from all experiments performed at three stages of maturation, with the median, 10^th^, 25^th^, 75^th^ and 90^th^ percentiles depicted as vertical boxes with error bars. *, P<0.001 compared with the relevant isotype control values for each clone as determined by one way analysis of variance (which is a subset of all control values depicted at each time point). All antibodies were tested at each time point at least three times except for CD82 423524 (twice on day 5, once on day 12), CD81 1D6 (thrice on day 8, once on day 12) and CD81 JS81 (thrice on day 12). Coating concentrations of fibronectin FnIII_12-IIICS-15_ (H/120) were 2.00 µg/ml, 1.25 µg/ml and 1.00 µg/ml for ProEB, BasoEB and PolyEB, respectively.
(TIF)
