# Supplementary figures and images for: Tetraspanins CD81 and CD82 Facilitate α4β1-Mediated Adhesion of Human Erythroblasts to Vascular Cell Adhesion Molecule-1
Source: PLoS One. 2013 May 21;8(5):e62654. doi: 10.1371/journal.pone.0062654 (PMC3660455; doi:10.1371/journal.pone.0062654)

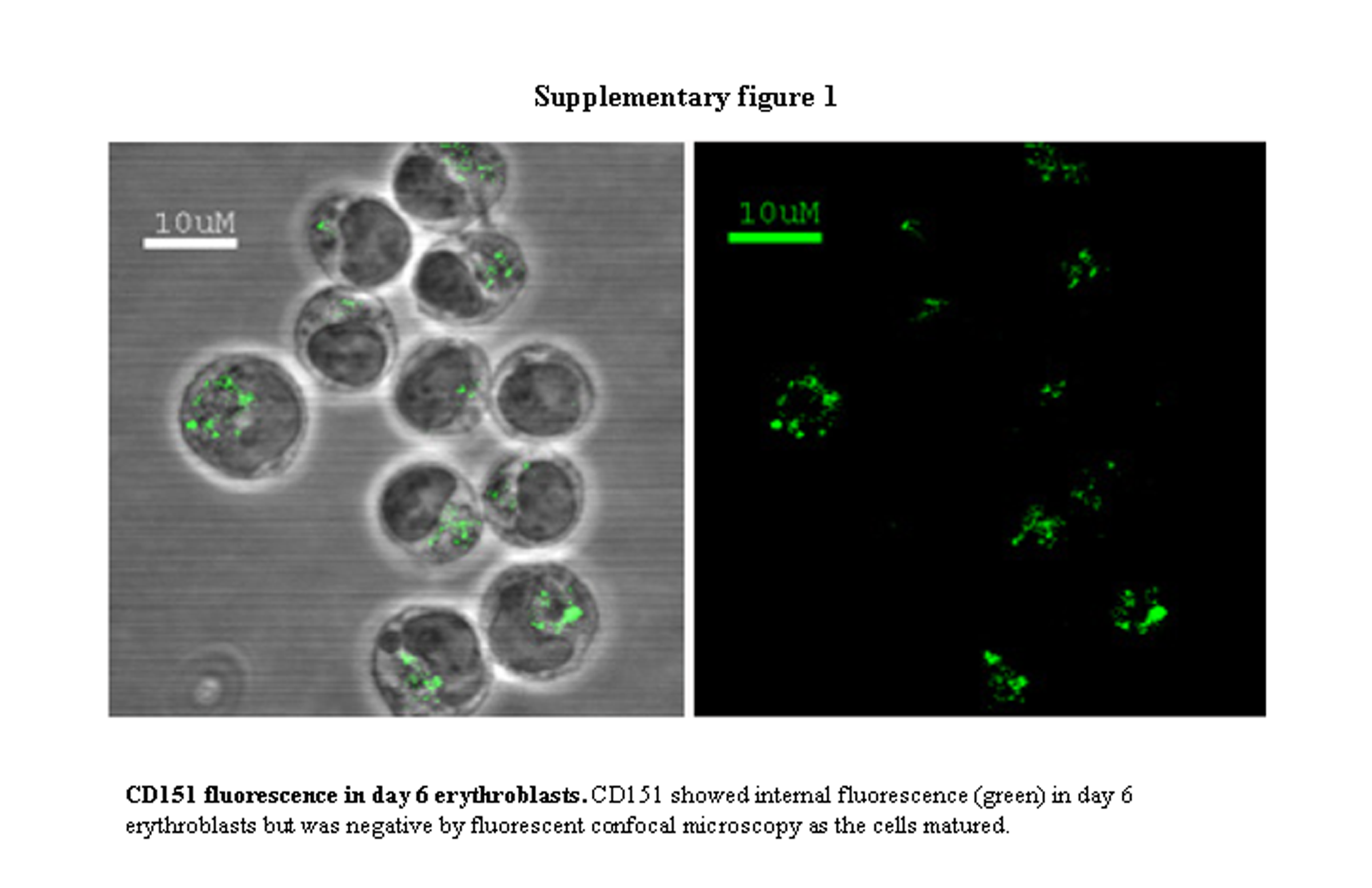

Supplement: Figure S1 — CD151 fluorescence in day 6 erythroblasts. CD151 showed internal fluorescence (green) in day 6 erythroblasts but was negative by fluorescent confocal microscopy as the cells matured (data not shown). (TIF) [file pone.0062654.s001.tif]

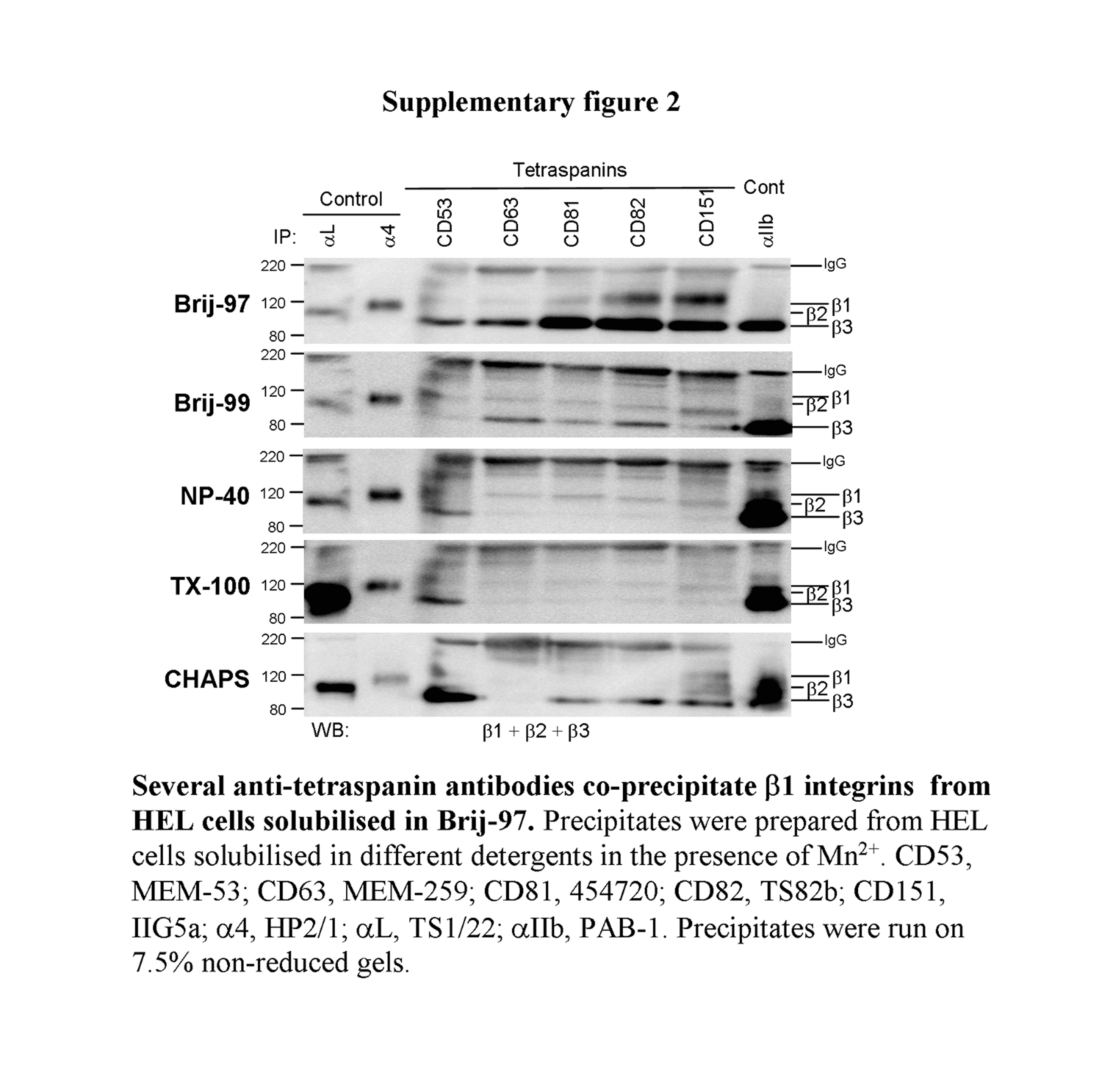

Supplement: Figure S2 — Several anti-tetraspanin antibodies co-precipitate β1 integrins from HEL cells solubilised in Brij-97. Precipitates were prepared from HEL cells solubilised in different detergents in the presence of Mn2+. CD53, MEM-53; CD63, MEM-259; CD81, 454720; CD82, TS82b; CD151, IIG5a; α4, HP2/1; αL, TS1/22; αIIb, PAB-1. Precipitates were run on 7.5% non-reduced gels. (TIF) [file pone.0062654.s002.tif]

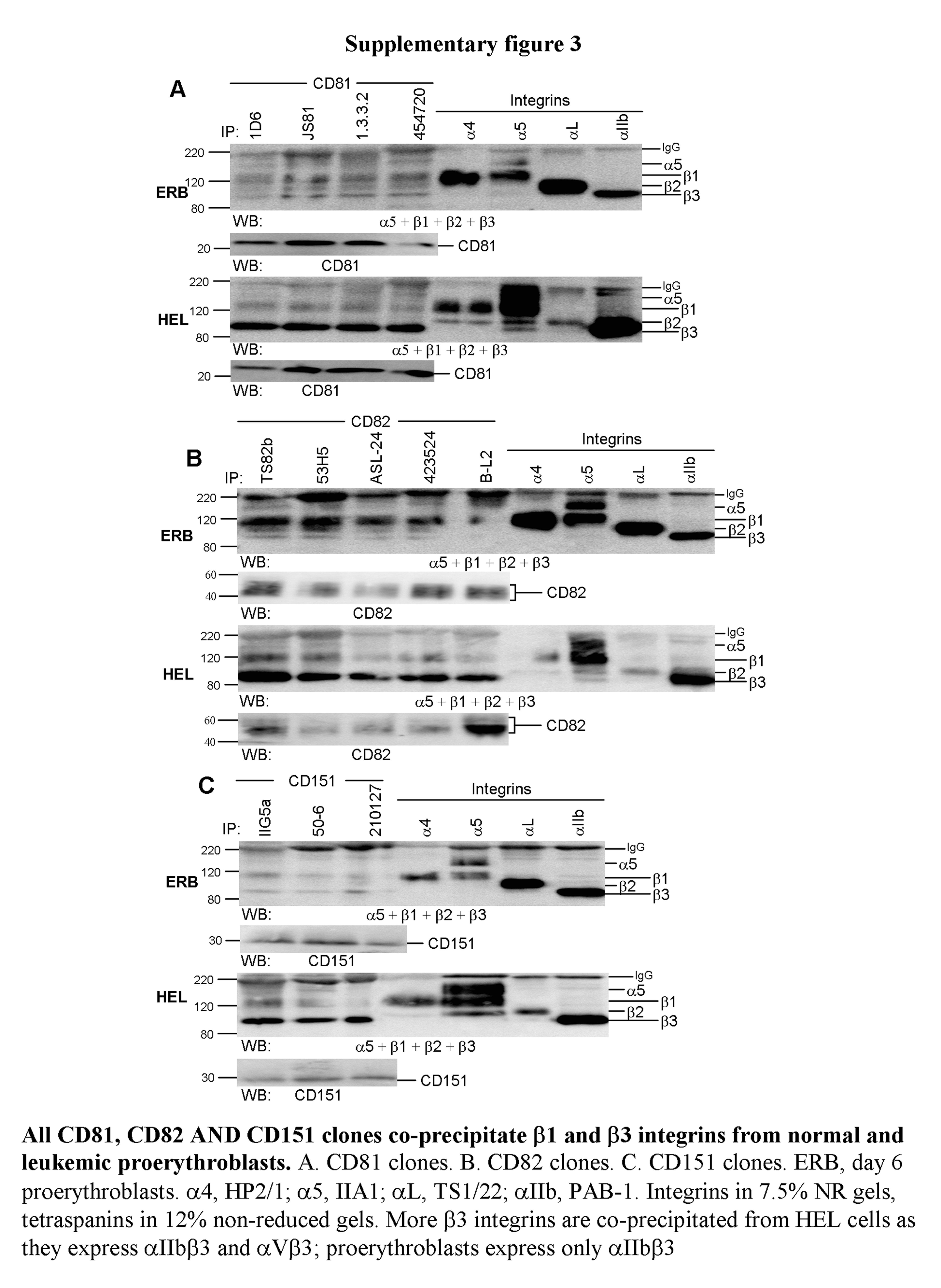

Supplement: Figure S3 — All anti-CD81, anti-CD82 and anti-CD151 clones co-precipitate β1 and β3 integrins from normal and leukemic proerythroblasts. A. CD81 clones. B. CD82 clones. C. CD151 clones. ERB, day 6 proerythroblasts. α4, HP2/1; α5, IIA1; αL, TS1/22; αIIb, PAB-1. Integrins in 7.5% NR gels, tetraspanins in 12% non-reduced gels. More β3 integrins are co-precipitated from HEL cells as they express αIIbβ3 and αVβ3; proerythroblasts express only αIIbβ3. (TIF) [file pone.0062654.s003.tif]

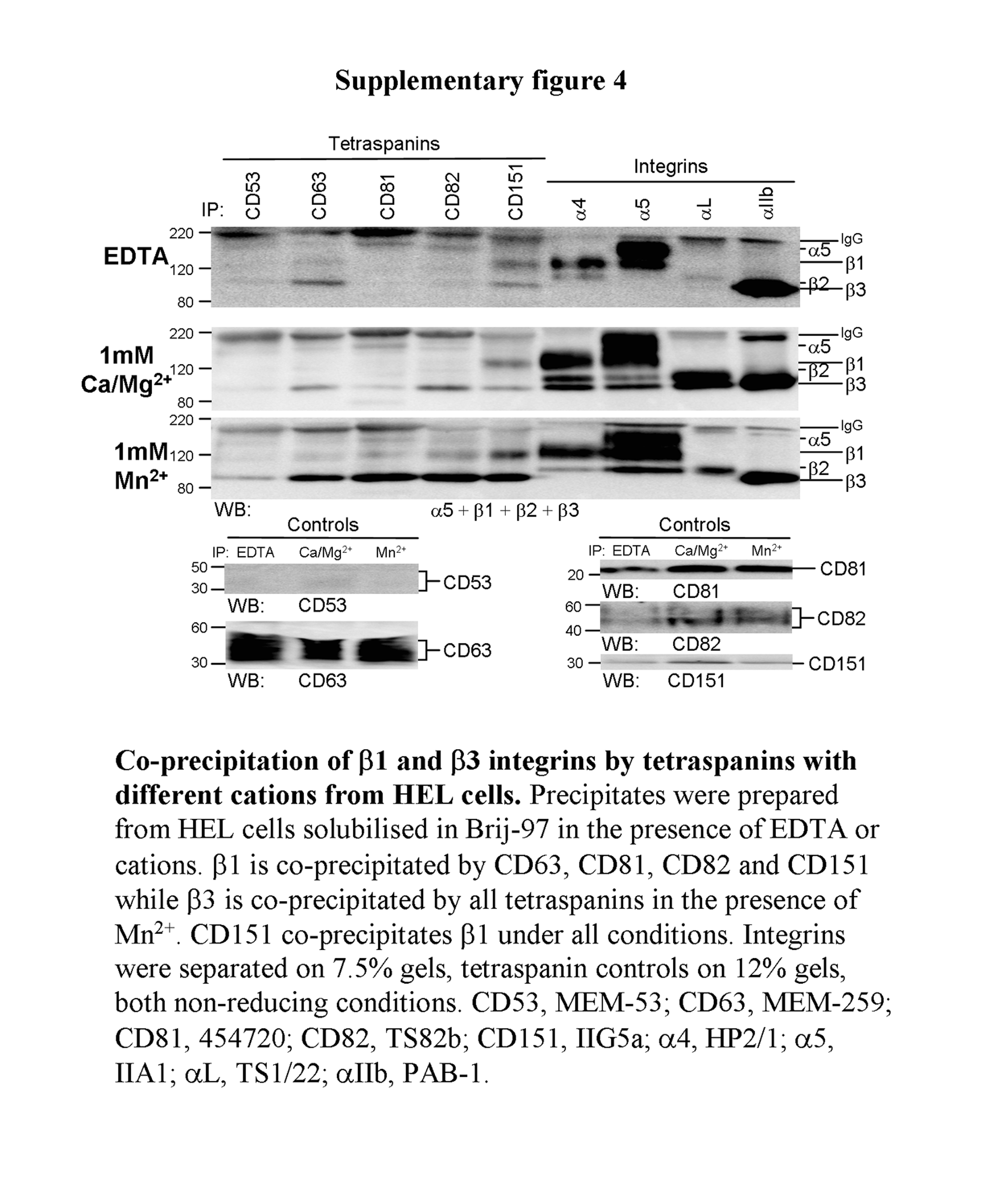

Supplement: Figure S4 — Co-precipitation of β1 and β3 integrins by tetraspanins with different cations from HEL cells. Precipitates were prepared from HEL cells solubilised in Brij-97 in the presence of EDTA or cations. β1 is co-precipitated by CD63, CD81, CD82 and CD151 while β3 is co-precipitated by all tetraspanins in the presence of Mn2+. CD151 co-precipitates β1 under all conditions. Integrins were separated on 7.5% gels, tetraspanin controls on 12% gels, both non-reducing conditions. CD53, MEM-53; CD63, MEM-259; CD81, 454720; CD82, TS82b; CD151, IIG5a; α4, HP2/1; αL, TS1/22; αIIb, PAB-1. (TIF) [file pone.0062654.s004.tif]

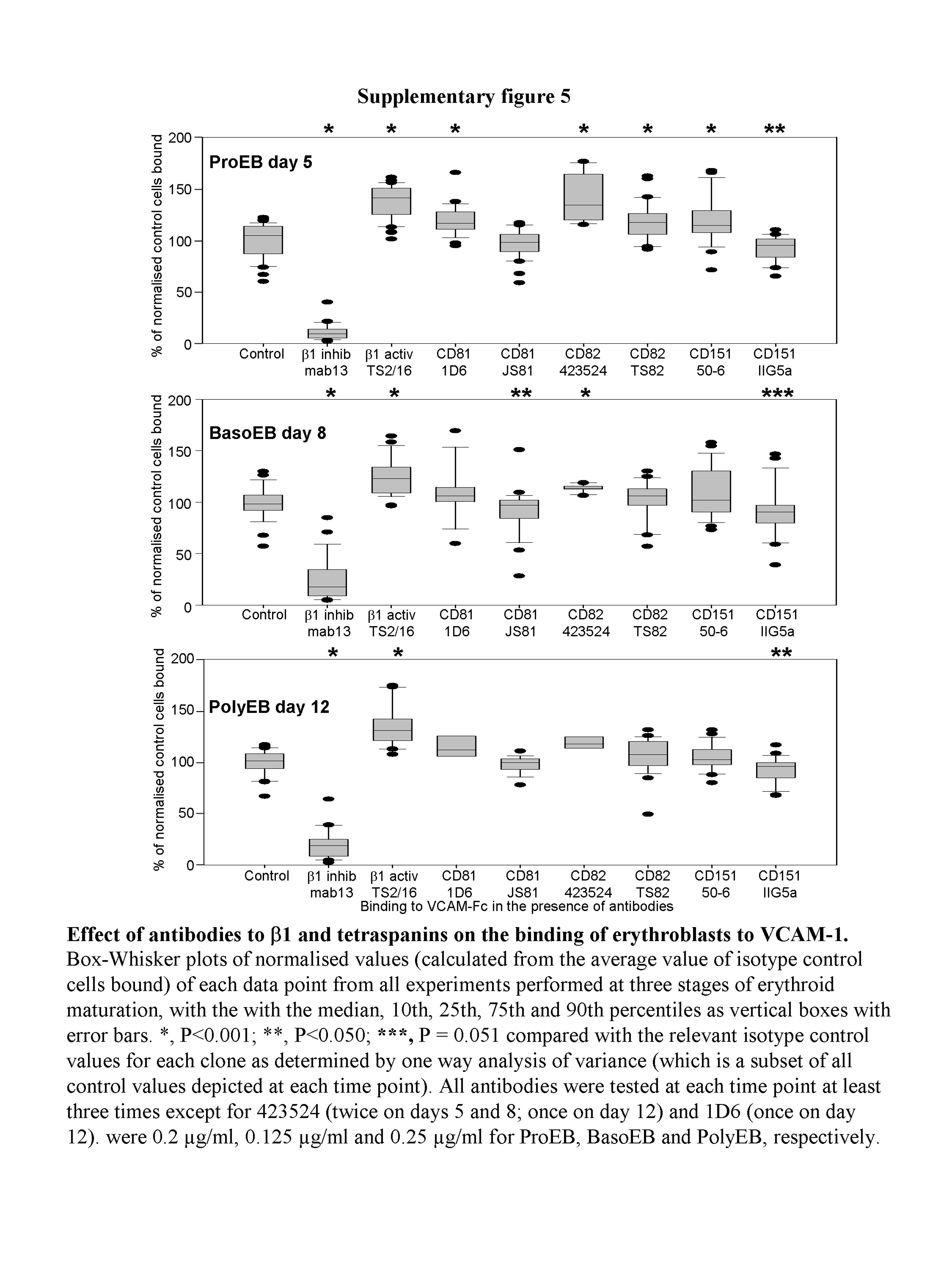

Supplement: Figure S5 — Effect of antibodies to β1 and tetraspanins on the binding of erythroblasts to Vascular Cell Adhesion Molecule-1. Box-Whisker plots of normalized values (calculated from the average value of isotype control cells bound) of each data point from all experiments performed at three stages of maturation, with the median, 10th, 25th, 75th and 90th percentiles depicted as vertical boxes with error bars. *, P<0.001, **, P<0.050; ***, P = 0.051, compared with the relevant isotype control values for each clone as determined by one way analysis of variance (which is a subset of all control values depicted at each time point). All antibodies were tested at each time point at least three times except for CD82 423524 (twice on days 5 and 8, once on day 12) and CD81 1D6 (once on day 12). Coating concentrations of VCAM-1Fc were 0.2 µg/ml, 0.125 µg/ml and 0.25 µg/ml for ProEB, BasoEB and PolyEB, respectively. (TIF) [file pone.0062654.s005.tif]

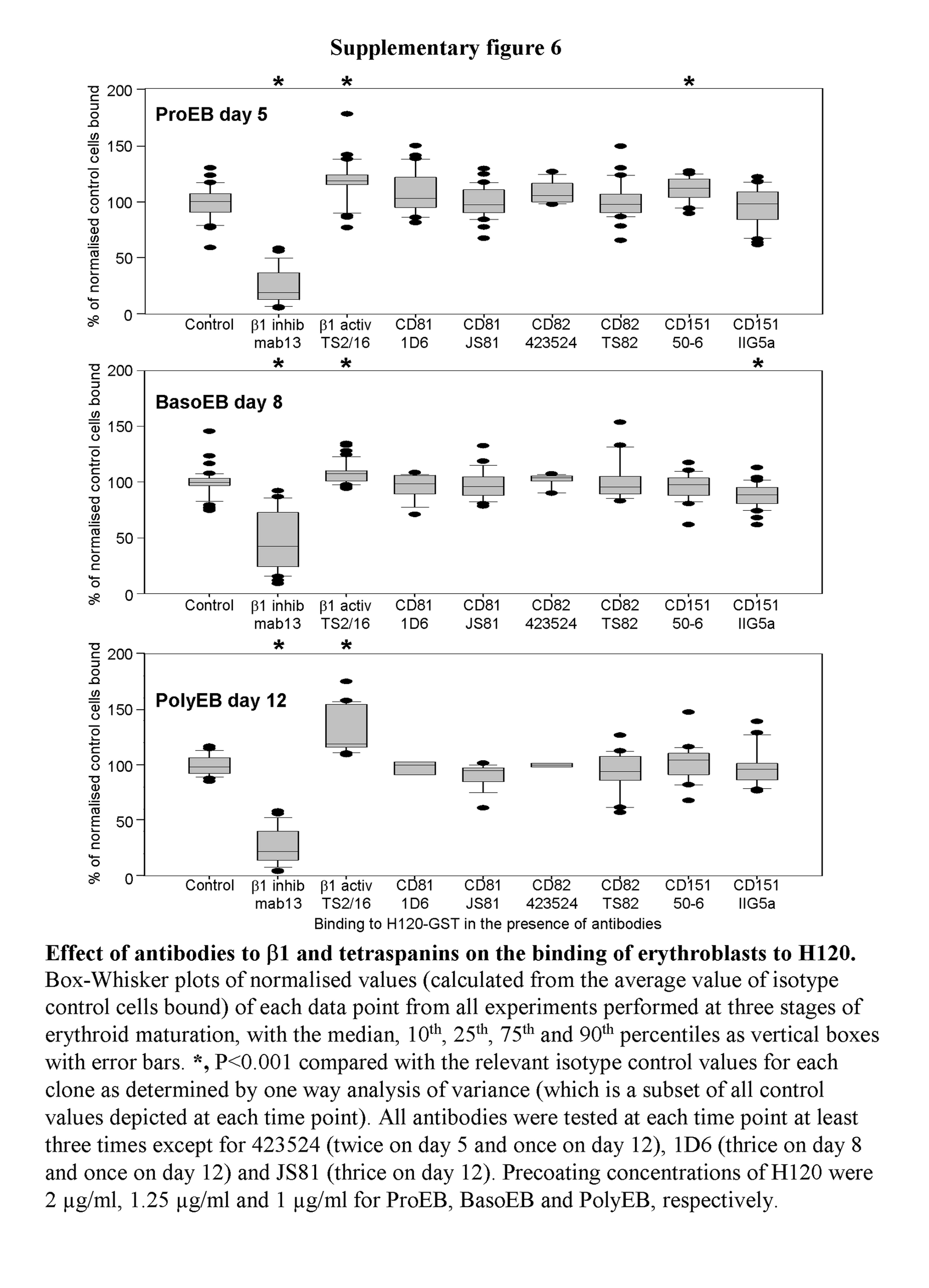

Supplement: Figure S6 — Effect of antibodies to β1 and tetraspanins on the binding of erythroblasts to fibronectin fragment FnIII12-IIICS-15. Box-Whisker plots of normalized values (calculated from the average value of isotype control cells bound) of each data point from all experiments performed at three stages of maturation, with the median, 10th, 25th, 75th and 90th percentiles depicted as vertical boxes with error bars. *, P<0.001 compared with the relevant isotype control values for each clone as determined by one way analysis of variance (which is a subset of all control values depicted at each time point). All antibodies were tested at each time point at least three times except for CD82 423524 (twice on day 5, once on day 12), CD81 1D6 (thrice on day 8, once on day 12) and CD81 JS81 (thrice on day 12). Coating concentrations of fibronectin FnIII12-IIICS-15 (H/120) were 2.00 µg/ml, 1.25 µg/ml and 1.00 µg/ml for ProEB, BasoEB and PolyEB, respectively. (TIF) [file pone.0062654.s006.tif]
